# Pulmonary lymphoid tissue induced after SARS-CoV-2 infection in rhesus macaques

**DOI:** 10.3389/fimmu.2025.1533050

**Published:** 2025-03-12

**Authors:** Zhong-Min Ma, Katherine J. Olstad, Koen K. A. Van Rompay, Smita S. Iyer, Christopher J. Miller, J. Rachel Reader

**Affiliations:** ^1^ California National Primate Research Center, University of California (UC) Davis, Davis, CA, United States; ^2^ Department of Pathology, Microbiology, and Immunology, School of Veterinary Medicine, University of California (UC) Davis, Davis, CA, United States; ^3^ Center for Immunology and Infectious Diseases, University of California (UC) Davis, Davis, CA, United States

**Keywords:** pulmonary mucosa associated lymphoid tissue, respiratory viral infection, SAR-CoV-2, immune response, CD169, animal model

## Abstract

**Introduction:**

Lung diseases are widespread worldwide. Pulmonary immunity plays a vital role against lung pathogens, including SARS-CoV-2 infection. Understanding the pathogenesis, including the development of local immune responses to infection, is fundamental for developing interventions to control the viral infection.

**Methods:**

Using immunohistochemistry, we investigated the distribution of immune cells in the lungs of rhesus macaques experimentally infected with SARS-CoV-2 and euthanized 11–14 days later.

**Results:**

Tertiary lymphoid tissue was found in all SARS-CoV-2 infected animals. The number (13.9 vs 1.5 iPLT number/ lung cm^2^), size (25992 vs 13946 µm^2^) and total area (0.46 vs 0.02 mm^2^ iPLT/ lung cm^2^) of the lymphoid tissue aggregations were significantly higher in SARS-CoV-2 infected animals than that of normal controls. This induced pulmonary lymphoid tissues comprised B cells, T cells, CD169 macrophages, and follicular dendritic cells with evidence of lymphocyte priming and differentiation.

**Discussion:**

The results suggest local immunity plays an important role in the SARS-CoV-2 infection. Further study of pulmonary immunity could lead to new interventions to develop vaccine strategies and discover new immune-regulatory biomarkers in monitoring and controlling SARS-CoV-2 infection and other lung diseases.

## 1 Introduction

Lung diseases are widespread worldwide, and the recent COVID pandemic has highlighted the importance of understanding the initiation and development of pulmonary immunity. The pulmonary immune system plays a critical role not only in preventing disease but also in the development of disease. While vaccines have been relatively successful in controlling the SARS-CoV-2 pandemic, a big challenge is the continuous emergence of variants of concern with unpredictable virulence and variable impact on vaccine efficacy (1). Above all, understanding the pathogenesis, including the development of local immune responses to infection, is fundamental for developing interventions to control the viral infection.

Classical pathology investigations, such as postmortem examination and histology, address initial questions about the pathogenesis of SARS-CoV-2 infection. However, human materials from COVID-19 patients that can be used for pathological studies are limited. Currently, pathological knowledge about COVID-19 comes from some postmortem biopsies and a limited number of autopsies (2). All these materials represent the latest stage of the disease, and only less than 5% of infected individuals reached this stage (3–7). Currently, the fatality rate of COVID-19 have dropped significantly. Furthermore, human sample collection usually happens more than 24 hours after death, which significantly limits additional assays and data quality. Therefore, animal models play a valuable role in studying pathogens and the prognosis of SARS-CoV-2 infection.

Several non-human primate species (NHP), including rhesus and cynomolgus macaques and African green monkeys, are helpful animal models of SARS-CoV-2, as infection mimics human SARS-CoV-2 infection in many aspects of the disease (8), including asymptomatic or mild clinical symptoms in the majority of infections, but with histological lesions consistent with mild to moderate interstitial pneumonia (9, 10). Knowledge gained by studying NHP should assist in understanding the disease, testing the efficacy of vaccination and treatment, studying sequelae following SARS-CoV-2 infection, and guiding in developing strategies for defeating COVID-19.

Currently, pulmonary immunity is referred to the inducible bronchus-associated lymphoid tissue (iBALT). It is a tertiary lymphoid organ that forms in the lung following inflammation or infection and is located at the basal site of the bronchial epithelium and perivascular space (11–13). In this paper, we use inducible pulmonary lymph tissue (iPLT) to emphasize the nature of pulmonary immunity, since not all of this tissue is associated with bronchi, lower levels of the airways, including alveoli, are potential sites of exposure to immunogens and development of immune reactions and iPLT formation. Unlike secondary lymphoid organs, such as the spleen and lymph node, the iPLT is not encapsulated and does not constitutively exist in all mammals including mice, monkeys, and humans (13–16). It is induced by exposure to antigens under infection or inflammatory stimulation (17, 18). IL-1 alpha, IL-23, IL-17, IL-22, CCL19, CCL21, CXCL12, and CXCL13 and their receptors such as CCR7, CXCR4, and CXCR5 play essential roles in iPLT’s formation and development (11–13, 19). The iPLT is characterized by a cluster of B cells, T cells, and follicular dendritic cells, which organize into a unique structure resembling lymphoid follicle (19). Although iPLT has not been extensively studied, there is evidence that it plays both a protective and pathological role in pulmonary disease (11, 12). The development of iPLT has not previously been systemically reported in COVID-19.

In this study, we investigated lungs of rhesus macaques harvested from 11-14 days post SARS-CoV-2 inoculation and utilized histopathology and immuno-histochemistry to characterize the histopathological lesions and the distribution of immune cells. Results showed that iPLT was well established in all experimental animals. The number (13.9 vs 1.5 iPLT number/lung cm^2^), size (25992 vs 13946 µm^2^) and total area (0.46 vs 0.02 mm^2^ iPLT/lung cm^2^) of the lymphoid tissue aggregations were significantly bigger in SARS-CoV-2 infected animals than that of normal controls. The iPLT is composed of B cells, T cells, and follicular dendritic cells with evidence of lymphocyte priming and differentiation. Our results suggested that local immunity may play an essential role in the pathogenesis of COVID-19. Further study of local immunity in the lung is necessary and would benefit understanding SARS-CoV-2 pathogenesis and could lead to new interventions to control the SARS-CoV-2 infection and disease.

## 2 Materials and methods

### 2.1 Animal information

Detailed information on the study’s experimental design was reported earlier (20, 21). Briefly, this study included 5 males and 6 females colony-bred Indian-origin rhesus macaques (*Macaca mulatta*) with ages ranging from 4 to 5 years old and weights ranging from 5.4 to 10.7 kg (median 8.6 kg). All animals in the study were born and raised in the California National Primate Research Center (CNPRC) breeding colony. The CNPRC is accredited by the Association for Assessment and Accreditation of Laboratory Animal Care International (AAALAC). Animal care complied with the 2011 *Guide for the Care and Use of Laboratory Animals* provided by the Institute for Laboratory Animal Research. All animals are negative for SARS-CoV-2, type D retrovirus, SIV, and simian lymphocyte tropic virus type 1. The study was conducted in a biosafety level 3 (ABSL-3) facility. Macaques were housed indoors in stainless steel cages (Lab Product, Inc.) as per national standards. For virus inoculation and nasal secretion sample collection, animals were anesthetized with 10 mg/kg ketamine hydrochloride injected i.m. and 15−30 µg/kg dexmedetomidine HCl injected i.m. Anesthesia was reversed with 0.07–0.15 mg/kg atipamezole HCl injected i.m.

Animals were inoculated with 1.2 × 106 PFU/ml (corresponding to 2 × 109 vRNA) California isolate of SARS-CoV-2 (SARS-CoV-2/human/USA/CA-CZB59×002/2020: GenBank accession number MT394528) by intratracheal, intranasal, and ocular routes. Viral RNA and SARS-CoV-2antibodies were monitored to confirm the SARS-CoV-2 infection. Three healthy animals with matching age and weight were included as uninfected controls. The UC-Davis Institutional Animal Care and Use Committee approved the study (study protocol 21735).

### 2.2 Pathology and immune-histochemistry

Animals were euthanized with an overdose of pentobarbital at 11 to 14 days after the viral inoculation. Lungs were infused gently with 10% buffered formalin within 20 minutes of harvestat necropsy. Infused lungs were further fixed in 10% buffered formalin for 3 days, then transferred into 70% ethanol and processed to make paraffin blocks. Four µm sections were used for Hematoxylin-Eosin (HE) and immunohistochemistry (IHC) stains. Resources and the working dilution of antibodies are shown in **Table 1**. All first antibodies were subjected to an antigen retrieval step consisting of incubation in AR10 (Biogenex, San Ramon, CA) for 2 minutes at 125^0^C in the Digital Decloaking Chamber (Biocare, Concord CA) which was followed by cooling to 90^0^C for 10 minutes, or incubation in antigen unmasking solution H3300 (Vector, Burlingame, CA) for 20 minutes at 100^0^C before rinsing in water (**Table 1**). EnVision and AEC (Dako, Santa Clara, CA) were the detection systems used. Slides were counterstained with hematoxylin, dehydrated, cover-slipped, and visualized using a bright field microscope. In the immunofluorescent stains of CD3/PD-1 and CD3/CD20/Bcl-1, Goat anti-Rat Alexa Fluor 488 and Goat anti-Rabbit Alexa Fluor 568, and Goat anti-Rat Alexa Fluor 488, Goat anti-Rabbit Alexa Fluor 647 and Goat anti-Mouse Alexa Fluor 568 (Invitrogen, Rockford IL) were used. Primary antibodies were replaced by normal rabbit IgG, mouse IgG and rat IgG (Invitrogen, Rockford IL) and included in each staining series as the negative control. All negative controls worked correctly (**Supplementary Figure S1**). With appropriate filters, slides were visualized with epi-fluorescent illumination using a Zeiss Imager microscope (Carl Zeiss Inc., Thornwood, NY).

**Table 1:** Antibodies’ resources and working conditions.

Ab name	Species	Clone	Vender	Cat#	Dilution	Antigen Retrieval Method
CD3	Rabbit		DAKO, Santa Clara CA	A-0452	1:100	AR10
CD3	Rat	CD3-12	abcam, Wattham MA	Ab11089	1:100	H3300/AR10
CD4	Mouse	1F6	abcam, Wattham MA	ab846	1:25	AR10
CD8	Rabbit		DBS, Pleasanton CA	RP-100	1:50	AR10
CD20	Mouse	L26	DAKO, Santa Clara CA	M0755	1:300	AR10
CD20	Rabbit		DAKO, Santa Clara CA	A-0452	1:100	AR10
MPO	Rabbit		abcam, Wattham MA	ab9535	1:50	AR10
CD68	Mouse	KP1	Thermo Fisher, Tremont CA	MS-397PO	1:100	AR10
CD163	Mouse	10D6	Novus, Centennial CO	NB110-59935	1:40	H3300
CD169	Rabbit	SP213	LSBio, Seattle WA	LS-C210436	1:100	H3300
Caspase 3	Rabbit	C92-605	BD, Franklin Lakes NJ	559565	1:200	AR10
Ki67	Rabbit		Neomarkers, Fremont CA	RB1510	1:300	AR10
IgG	Rabbit		DAKO, Santa Clara CA	A0423	1:1000	AR10
Perforin	Mouse		Mabtech, Cincinnati, OH	Pf-16:17	1:100	AR10
Granzyme B	Mouse	GZB01	Neomarkers, Fremont CA	MS-1157-R7	Prediluted	AR10
PD1	Rabbit		Novus, Centennial CO	NBP1-88104	1:100	H3300
Bcl-6	Mouse	LN22	Biocare, Concord CA	CM 410 A	1:100	AR10
p55	Mouse	55K-2	DAKO, Santa Clara CA	M3567	1:200	AR10

**Figure 1:**
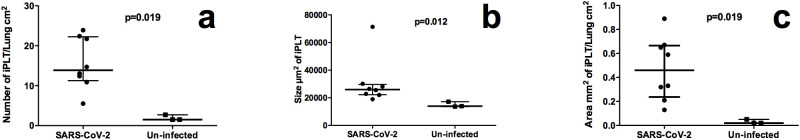
Quantity comparison of iPLT between SARS-CoV-2 infected and normal monkeys. **(a)**, SARS-CoV-2 infected monkeys had significant (p<0.05) more iPLTs. **(b)**,The size of iPLT was bigger in SARS-CoV-2 infected(p<0.05). **(c)**,Total area of iPLT were larger than that of normal monkeys (p<0.05).

To quantify the number and size of lymphoid tissue, lung sections were stained with CD3, CD20, and CD169 antibodies and Goat anti-Rat Alexa Fluor 488, Goat anti-Rabbit Alexa Fluor 647 and Goat anti-Mouse Alexa Fluor 568 (Invitrogen, Rockford IL). The whole section was scanned using a Lecia DM6 microscope and Leica LAS X software. NIH Image J was used to measure the lymphoid tissue. CD3, CD20, and CD169 positive cell aggregations were outlined (**Figure 1**) when the total cell number was more than 30 (around 5000µm^2^). The number and area of the cell aggregations were reported using the area of the lung (square center meter, cm^2^) as a reference.

### 2.3 Statistical analysis

**Figure 2:**
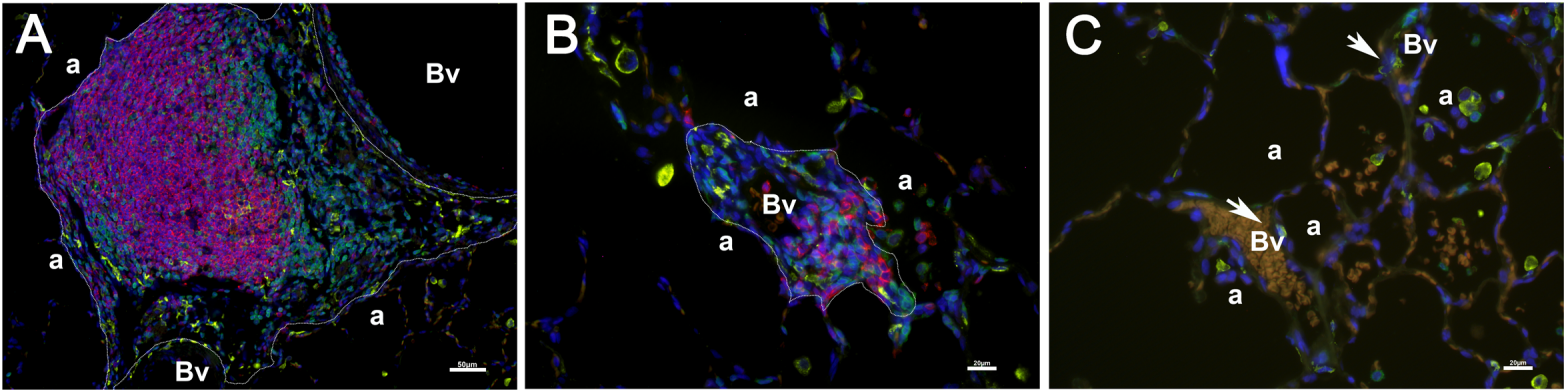
Aggregations of CD3 (green), CD20(red), and CD169 (yellow) positive cells were used to identify the number and size of iPLT. Two iPLTs were outlined **(a, b)**. Additionally, blood vessels (Vs) and alveoli (a) were labeled. No bronchi were seen within the iPLT regions. A few CD169 positive cells (pointed by arrow) surrounded blood vessels **(c)**. Scale bar on **(a)** is 50µm. Scale bars on **(b, c)** is 20 µM.

Statistical analysis was performed using Prism software (GraphPad Software, Boston, MA). Resuls were reported as median. Median and interquartile range of results shown on **Figure 2**. Non-parametric Mann-Whitney test was applied to compare SARS-CoV-2 infected animals with uninfected controls.

## 3 Results

### 3.1 General histopathology findings

As described earlier (20, 21), all animals were infected after SARS CoV-2 inoculation. No animals showed severe clinical signs (such as acute fever, weight loss, or respiratory distress) after infection. Clinical signs were absent or mild.

Detailed histopathological findings had been reported (22). Briefly, gross examination showed the pleural surface of the lungs was smooth with randomly distributed, variable-sized, and irregular-shaped dark or pale discolorations. No pleural effusion was seen. Enlargement of lymph nodes at the hilum and mediastinum was noticed in some animals. In cross-sections, small patches of the discolorations were randomly distributed in different lobes of the lungs with evidence of mild pulmonary edema. Histopathological examination of the lungs showed multifocal to locally extensive interstitial pneumonia of mild severity, frequently radiating out from the terminal bronchioles and sometimes subpleural in all animals. Histology in these regions showed that expanded alveolar septa contained few to moderate numbers of neutrophils and mononuclear cells. Alveolar spaces variably contained macrophages, a few neutrophils, and occasional cellular debris. In some areas, there was occasional type 2 pneumocyte hyperplasia. Perivascular cuffing by inflammatory cells was present to variable degrees in all animals.

### 3.2 Immunohistochemistry results

#### 3.2.1 Pulmonary lymph tissue increases after SARS CoV-2 infection

Using aggregations composed of CD3, CD20, and CD169 positive cells, we quantified the iPLT. The iPLT is evenly distributed in the lung, including the sub-pleura. The number (13.9 vs 1.5 iPLT number/lung cm^2^, **Figure 2a**), and size (25992 vs 13946 µm^2^, **Figure 2b**) of the lymphoid tissue aggregations were significantly higher in SARS-CoV-2 infected animals than that of uninfected control monkeys (p<0.05). The total area (0.46 vs 0.02 mm^2^ iPLT/lung cm^2^, **Figure 2c**) of lymphoid tissue aggregations were significantly bigger in SARS-CoV-2 infected animals than that of uninfected controls(p<0.05). Some of the lymph tissue was associated with bronchi. However, some of the iPLT can be found around small arteries within the alveolar parenchyma and not associated with bronchi. Even through a lot of small aggregations less than 5000µm^2^ were not included in counting the number the iPLT, the total area and number of the lymph aggregations were significantly increased in SARS-CoV-2 infected animals (p<0.05, **Figure 2**).

#### 3.2.2 Characterization of Pulmonary lymph tissue

**Figure 3:**
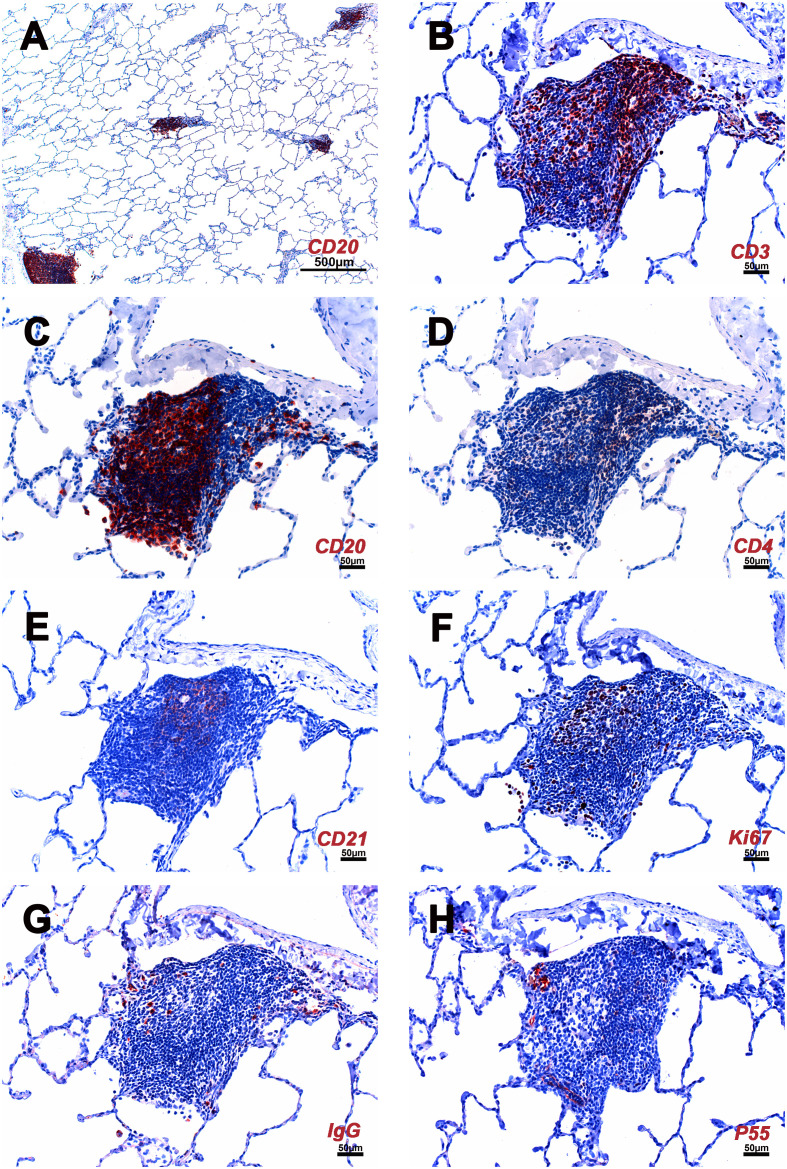
Cellular composition of iPLT of rhesus macaques infected with SARS CoV-2. **(A)** Multiple iPLTs identified by CD20 IHC stain. The size of the iPLTs was variable. **(B)** CD3 positive T cells aggregated along the periphery and scattered throughout the entirety of the iPLT. **(C)** CD20 positive B cells mimic a germinal center. **(D)** CD4 positive cells. **(E)** CD21 positive cells in the germinal center have a dendritic structure. **(F)** Ki67 (dividing cells) positive cells were seen in the germinal center. **(G)** IgG positive cells were found within the iPLT. **(H)** P55 positive cells were found in the iPLT, and some endothelial cells were identified, which formed well-defined small vessel structures. IHC-positive cells were in the red. The scale bar on panel **(A)** was 500µm. Scale bars on panels **(B–H)** were 50µm.

iPLT was found in all animals. In addition to well-developed lymphoid follicles with a germinal center associated with bronchi and bronchioles, a significant amount of the iPLT is located in the perivascular space. The iPLT was of variable size and was randomly distributed throughout the lung parenchyma (**Figure 3**). In some location, several aggregations clustered together near one blood vessel or bronchus. There clustered lymph aggregations could be larger than 1 mm^2^. More study is needed to confirm wheather this kind of structure is visible under CT scan or other examination methods.

**Figure 4:**
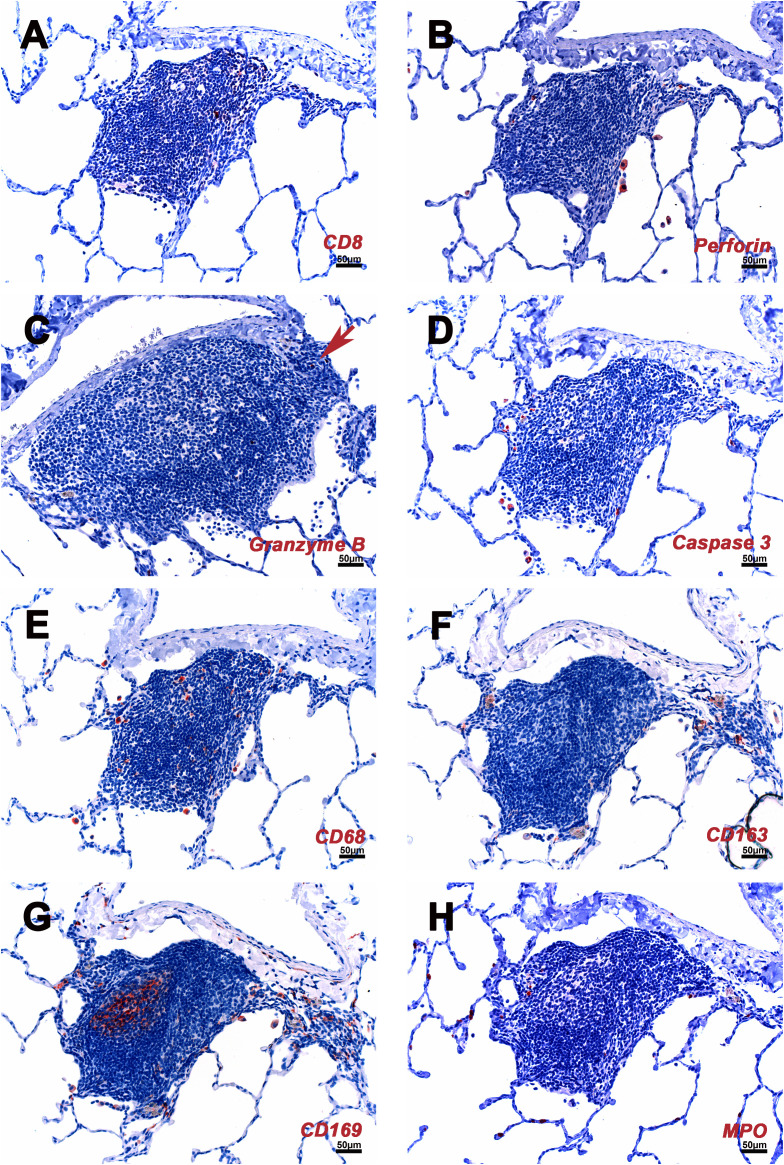
Cellular composition of iPLT of rhesus macaques which infected with SARS CoV-2. **(A)** Some CD8 positive cell are located in the T cells zone. **(B)** A few perforin positive cells found in iPLT and alveoli. **(C)** A few granzyme positive cells (arrow) were seen. **(D)** A few caspases 3 positive cells were found at the edge of iPLT and inside alveoli. **(E)** CD68 positive cells seen in iPLT and alveoli. **(F)** CD163 positive cells. **(G)** CD169 positive cells were found in the germinal center, border of alveoli and iPLT, and inside alveoli. **(H)** HPO positive cells. IHC-positive cells were in the red. Scale bars on all panels were 50µm.

**Figure 5:**
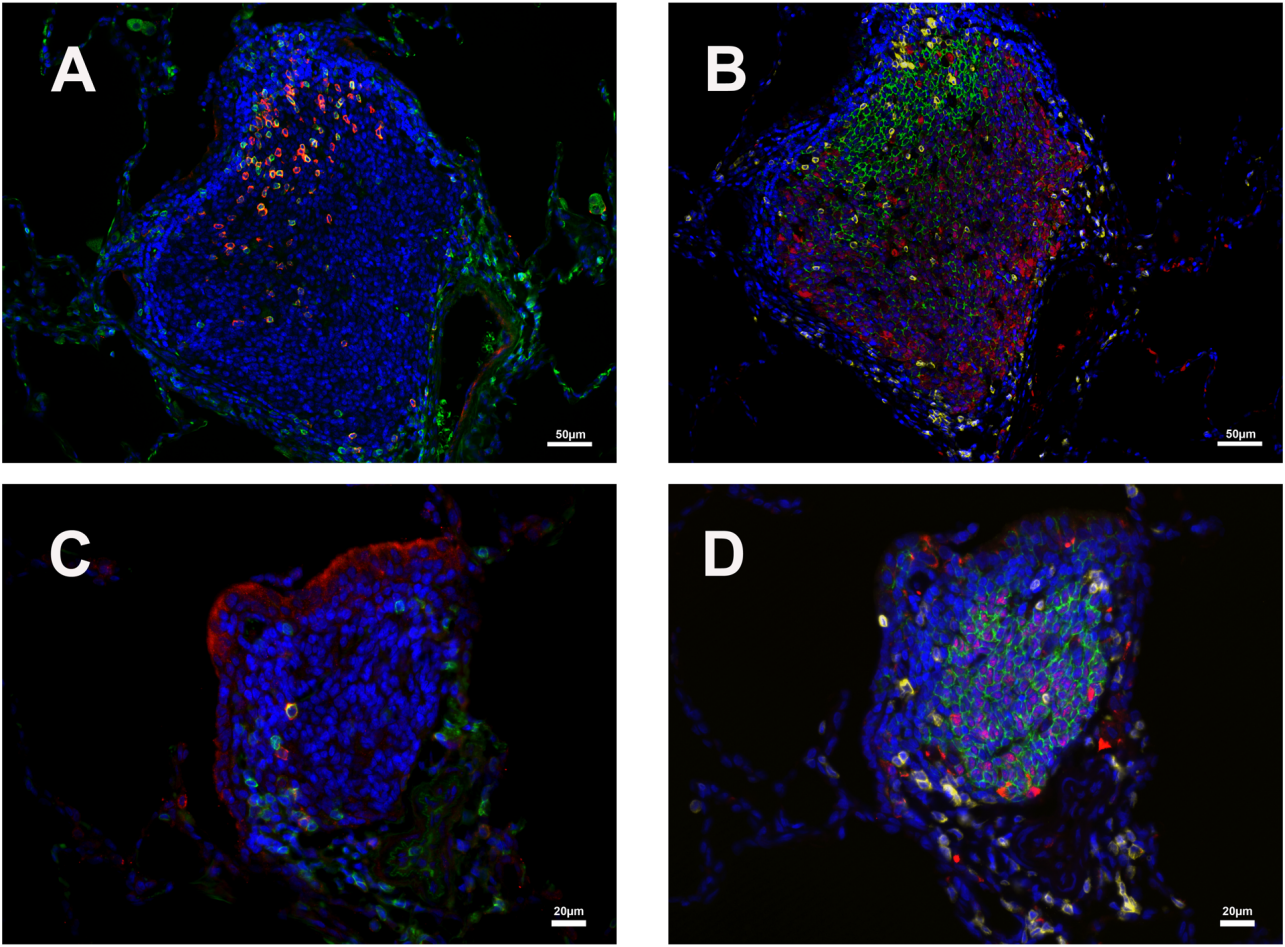
Distribution of CD3/PD1 (germinal center T_fh_ cells) and CD20/Bcl6 (germinal center B cells) double-positive cells in two iPLTs of different size. **(A, B)** One relative larger germinal center. **(C, D)** One relative smaller germinal center. **(A, C)** CD3 (green) and PD1 (red) double-positive cells in germinal center of iPLTs. **(B, D)** CD20 (green) and Bcl-6 (red) double-positive cell in B cell zone of iPLTs of different sizes. CD3 (yellow) positive cells are also shown. The scale bars on panels **(A, B)** were 50µm. Scale bars on panels **(C, D)** were 20µm.

The major constituents of the iPLT were CD20+ B cells. The B cells were arranged in prominent follicular structures, which were sometimes eccentric and sometimes had a germinal center. CD20 and Bcl6 double-positive cells, Ki67 positive cells, Caspase-3 positive cells, and CD21 follicular dendritic cells were noticed in the germinal center (**Figures 3**–**5**). CD20 and Bcl6 double-positive cells are germinal center B cells. BCL-6 expression is topographically restricted to germinal centers, including all centroblasts and centrocytes (23). Ki67 and Caspase-3 positive showed evidence of cell proliferation and program death in the germinal centers. CD3+ T cells, mostly CD4+, were scattered within the B cell zone or polarized at one edge of the iPLT (**Figure 3**). A few CD8+ cells were present throughout the iPLT (**Figure 4**). We used CD3 and PD-1 double-positive cells to identify T follicular helper cells(Tfh), which play a crucial role in specific adaptive immunity development and maturation (24, 25). Some of the CD3+ cells were PD-1 positive and were found within germinal centers(**Figure 5**). This evidence supports lymphocyte priming, differentiation and indicates B-cells proliferate and undergo affinity maturation to develop high-affinity antibodies in the iPLTs.

CD68, CD163, and CD169 positive macrophages were examined in the current study. Most of the CD163+ and CD68+ cells were at the edge of the B cell zone, while CD169+ cells were also found at the center of the germinal center (**Figures 1**, **4**). Solid positive strong CD169+ cells in the center of the germinal center and outer margin, especially between alveoli and lymphoid follicules, which are similar to CD169 positive cells in other lymph tissues such as lymph node and spleen, allow the CD169 cells close contact with circulation and inhaled outside antigens (26)(**Figures 1**, **4**). Some spindle-shaped CD169+ cells also existed around larger blood vessels (**Figure 1**). CD169 staining was very weak in macrophages within the lumen of bronchi and within alveoli. There were numerous CD68 positive macrophages in the lumen of bronchi and inside the alveoli. Multinuclear CD68 cells were also found in the alveoli. Adjacent to the lymphoid aggregations, there were some macrophages with weak CD68 positive. Foreign materials were found in their cytoplasm. Those cells were also CD163, Caspase-3, and perforin positive. CD163+ and CD169+ cells, which were bigger than CD68+ cells, shared a similar distribution pattern with CD68+ cells beyond the iPLT areas of the lung (**Figures 1**, **4**).

A few IgG-positive cells were found within the iPLT (**Figure 3**). Further, in the iPLT that appeared as more homogeneous aggregates without classical B cell, T cell zone, and well-developed germinal center, relatively more Ki-67+ cells were found (**Figure 3**). This suggests that the classical iPLT originated from small aggregations of immune cells. Thus, an adaptive humoral immune response occurred in the lungs of monkeys infected with SARS-CoV-2.

The P55 stain identified some endothelial cells that formed well-defined small vessel structures inside the iPLT (**Figure 3**). Some CD21+ cells were found around small blood vessels where lymphocytes existed. Perforin and granzyme B + cells were occasionally seen in iPLT (**Figures 3**, **4**).

#### 3.2.3 Immune cells beyond the iPLT areas of the lung

We also investigated the distribution of immune cells in other parts of the lung, beyond the iPLT areas. Myeloperoxidase (MPO) positive neutrophils were found in blood vessels, alveolar walls, alveoli, and around small blood vessels. More MPO + cells were in the exudate within alveoli and focal areas with interstitial pneumonia (**Figure 4**). In a few cases, neutrophils infiltrated the depth of the bronchial wall to appear in the bronchial lumen. There were more MPO+ cells than CD3+ T cells, especially in the areas with interstitial pneumonia. CD3+ cells were evenly distributed in the lung with no significant difference in the number of these cells, regardless of pathology. They were found in areas away from interstitial pneumonia Most CD3+ cells were found around small blood vessels and formed aggregations in the space between blood vessels and alveoli, which colocalized with the perivascular cuffing on HE slides. Only a few CD3+ cells are located in the alveolar walls. In contrast to CD3+ T cells, CD20+ B cells were not only the primary element of iPLT but also frequently found inside alveoli and within alveolar walls. CD4 and especially CD8 staining was weak. As described previously, most CD4+ and CD8 + cells are found within iPLT, and even fewer are scattered in the alveolar walls.

Perforin + cells were found in all animals, most of which were macrophages located in alveoli. There were relatively more perforin+ cells within the areas with pathology. Granzyme B identified different cell populations than the perforin stain. Most granzyme B positive cells were found in the alveolar walls. The size of the granzyme B positive cells is much smaller than that of perforin positive cells (**Figure 4**).

## 4 Discussion

Pulmonary immunity is unique. In humans, non-human primates, and mice, iPLT is a tertiary lymphoid organ that does not exist at birth (14, 15, 27). iPLT resembles a variably sized secondary lymph follicle similar to that found in a lymph node composed of many B cells in the center with surrounding T cells. Unlike most secondary lymphoid organs such as lymph nodes and spleen, which develop embryonically without microbial stimulation or antigen exposure, BALT develops after hosts have been exposed to microbial agents or experienced pulmonary disease (14). BALT has been renamed as inducible bronchus-associated lymphoid tissue, iBALT, to reflect this property (13, 19). In humans, the frequency of iBALT is relatively higher in children compared to people over 20 years old (28, 29). The frequency of BALT is less than 5 per 10 histology sections in groups of young adult rhesus monkeys (13). As the name BALT indicates, early investigators considered that BALT was located within the lamina propria of bronchi and belonged to the category of mucosal-associated lymph tissue (MALT). However, recent studies showed that iBALT could also be found in the perivascular space, which may be easily ignored when the cluster of immune cells is small, or the examined lung section is not processed correctly (16, 30, 31).

The lung has a dual blood supply. The bronchial arteries deliver blood to the bronchial wall, and the pulmonary arteries supply the alveoli. A unique capillary bed surrounds the pulmonary arteries (32, 33). Thus, during immune reactions and inflammation, leukocytes can enter the lung through 2 routes with different adhesion molecules and vessel structure (30, 32, 33) The Perivascular space is an important site in immune reactions where capillaries, lymphatic vessels and nerves can be found (30, 34).The lung is exposed to the outside environment through inhalation and exhalation. The deposition of inhaled particles depends on their flow velocity and size (35). While immunogens can deposit at bronchi and induce an immune response, some small particles can also reach into alveoli and invoke immune activities in the perivascular space. As shown in this study, lymph tissues not only found around bronchi, but also in the perivascular space surrounded by alveoli. Thus, using inducible pulmonary lymphoid tissue, iPLT, to describe the nature of local immunity of the lung is more accurate than using iBALT. So, we suggest changing the name of iBALT to iPLT to emphasize that this system was a local immunity of the lung, not only associated with bronchi.

Studies show that iPLT is not simply an accumulation of immune cells. Instead, these newly formed lymphoid tissues make a new micro-environment that recruits immune cells and organizes them into a tertiary lymphoid tissue that supports lymphocyte priming and differentiation (11, 13, 19, 36). It is controversial whether or not iPLT plays a protective or pathogenic role when the host is infected by specific pathogens or under some disease conditions. However, some studies show that iPLT is vital in viral clearance and reducing inflammatory responses after a viral infection such as the influenza (37), repiratory syncytial virus (RSV) (38). Furthermore, iPLT induced by one pathogen can affect the response to a second, or unrelated pathogen (39–41). Using a mice model, people successfully induced immunity against two different influenza viruses, a mouse-adapted SARS-coronavirus or mouse pneumovirus infection, by inhaling Protein Cage Nanoparticle (PCN). Mice treated with the PCN show a significantly high survival rate, quick viral clearance, and accelerated viral-specific antibody production (40). Cross-protection between influenza vaccination and COVID-19 has recently been reported (42–44). In addition, once iPLT is formed, memory T cells can be maintained, and iPLT can persist for months on site (31). Thus, agents that establish iPLT can potentially benefit the host by controlling future infections. Since the COVID-19 pandemic, human population have been exposed to SARS-CoV-2. There is a high chance that most people have iPLT established. The pulmonary Immunity status of human population changed dramatically compared with that of the pre-COVID-19 pandemic. There is a high probability that the spectrum of lung disease, especially infectious disease, has shifted. Studying the iPLT could lead to new interventions to control infectious lung diseases, including SARS-CoV-2 infection and other diseases.

Like influenza virus-infected mice (19), our study shows that iPLTs are well developed after SARS-CoV-2 infection. They are scattered throughout the parenchyma of the lung, where they are found not only in the upper bronchi but also in the vascular space without associated bronchioles, in the peripheral lung. The size and shape of iPLT varies as may its structure. Some iPLTs with well-developed lymphoid follicles were found within the wall of the upper bronchi. When secondary follicles were present, they consisted primarily of centrally located B cells, predominantly CD20+ and less often Bcl-6 positive. T-lymphocytes forming a rim around the germinal center and less often scattered within B-cell zones were predominantly CD3+ and CD4+ and rarely PD-1 positive. There were Ki67, Caspase-3, and IgG-positive cells in the follicle. Dendritic cells and macrophages were also noticed within the follicle. This evidence supports the role of iPLT in lymphocyte priming and differentiation. Like the previous study, germinal centers presented in the mediastinal lymph node following SARS CoV-2 infection (20), and the germinal centers also existed in the iPLTs. Besides the iPLTs with a well-developed lymphoid follicle structure, many small iPLTs were located in the vascular space without any association with bronchioles in the peripheral lung. With H&E stains, these small iPLTs are easily interpreted as peri-vascular cuffs and even ignored, especially when the lung was not appropriately perfused. With IHC stains, these iPLTs were obvious and had a similar immunophenotype of CD20/BLC2+ B cells, CD3/PD-1/CD4 positive T cells, and few dendritic cells and macrophages, as described in the larger iPLT despite the lack of germinal centers.

CD169, or Siglec1 or Sialoadhesin (Sn), is a surface adhesion molecule on human myeloid cells. It is an inducible and continuously expressed cell surface molecule on mononuclear phagocyte immune cells, including macrophages, monocytes, and dendritic cells, among mammalian species (45, 46). Sn is most abundantly expressed by macrophage subsets occupying secondary lymphoid tissue such as the splenic marginal sinus/perifollicular region and the lymph node sup-capsular sinus at the borders of lymphoid tissue and circulating fluids. These cells have a role in pathogen capture and Activation of T and B cells and anti-pathogen immune response (26). This conserved positioning of CD169 positive cells strongly suggests they play a role in regulating the immune responses to pathogens and self-antigens that they encountered (26, 46).

CD169 expression is associated with respiratory viral disease, bacterial infection, autoimmunity disease, cancer, and organ transplant rejection (45). Recently studies show CD169 associated with SARS-CoV-2 infection (47, 48). Monocyte CD169 was strongly overexpressed in COVID-19 patients (48). It has been suggested as an early diagnostic marker in SARS-CoV-2 infection (26, 45, 49). Current studies show more CD169 positive cells in the alveoli, perivascular space, and lymphoid aggregations of SARS-CoV-2 infected monkeys. Macrophages can be infected by the virus (50). Some are located between alveoli and lymphoid aggregations, the borders between lymphoid tissue and alveolar space. Thus, CD169 macrophages play an important role in antigen capturing, antigen presentation, and forming lymphoid organization during SARS-CoV-2 infection. Analysis of the level of CD169 expression in bronchoalveolar lavage fluid would be significant in understanding pulmonary immunity against SARS-CoV-2 infection and other diseases.

Local immunity against SARS-CoV-2 infection, specifically iPLTs, has not been well characterized in humans partly because of the lack of samples, especially during the early stages of the disease. We do not know what role iPLT plays during the disease process. Most SARS-CoV-2 samples taken from infected humans are at autopsy when primary changes induced by the virus, such as iPLT, are obscured by a severe inflammatory background associated with mortality. As such, the role of iPLT in the disease course is currently not well defined. However, evidence shows that outcomes of SARS-CoV-2 infection vary within the different age groups of people and different species (51, 52). To shed further light on mechanisms behind those differences, it is worth exploring the role that local immunity, including iPLT, plays during SARS-CoV-2 infection, in particular, what constitutes a healthy iPLT response (associated with no or mild disease) and whether iPLT is involved in triggering a fulminant inflammatory response that leads to severe disease and mortality. Using flow cytometry or other methods to identify immune cell ratios and cell distribution in different conditions, collecting bronchoalveolar lavage (BAL) fluid or sputum samples from longitudinally over the course of disease to monitor local immunity, clarify whether the iPLTs induced by infection or vaccines are protective or pathogenic, and determine whether iPLT remains as memory immunity capable of protecting against reinfection with the same or different pathogens need to be addressed in future. This paper is descriptive. However, the findings of this study suggested that further insights may lead to the development of new interventions to prevent or treat SARS-CoV-2 infection and other diseases.

## Data Availability

The original contributions presented in the study are included in the article/**Supplementary Material**. Further inquiries can be directed to the corresponding authors.

## Supplementary material

Supplementary Figure 1 Negative controls for IHC stains. **(A)** Negative control for CD3,CD20, and CD169 stain. **(B)** Negative control for CD3 and PD1stain. **(C)** Negative control for CD20, Bcl 6 and CD3 stain. **(D)** Negative control for IHC stains using AEC as chromogne. Scale bars on **(A–C)** were 50µm. Scale bar on **(D)** was 100µm.
