# Supplementary figures and images for: Pulmonary lymphoid tissue induced after SARS-CoV-2 infection in rhesus macaques
Source: Front Immunol. 2025 Mar 12;16:1533050. doi: 10.3389/fimmu.2025.1533050 (PMC11937022; doi:10.3389/fimmu.2025.1533050)

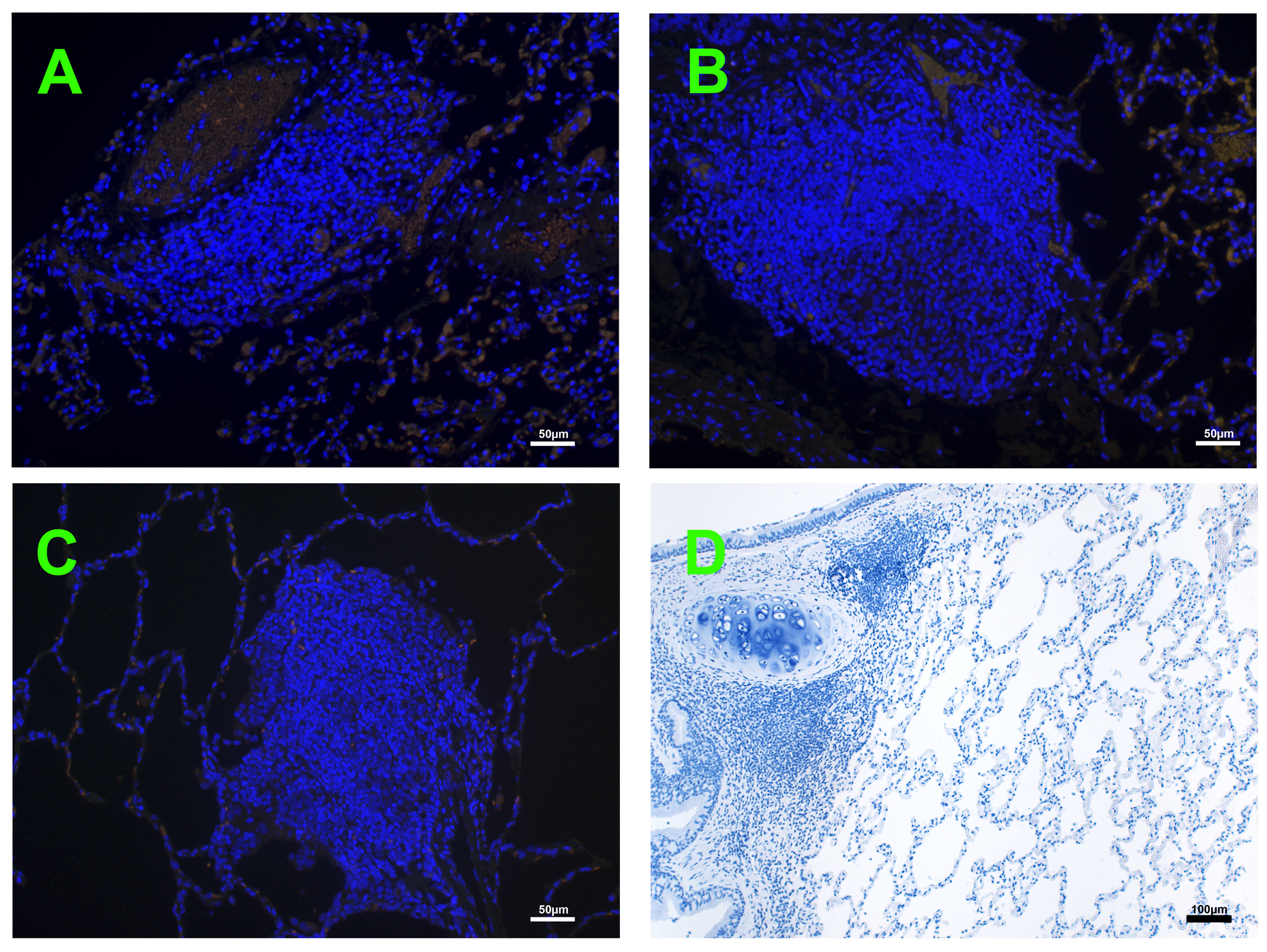

Supplement: Supplementary Figure 1 — Negative controls for IHC stains. (A) Negative control for CD3,CD20, and CD169 stain. (B) Negative control for CD3 and PD1stain. (C) Negative control for CD20, Bcl 6 and CD3 stain. (D) Negative control for IHC stains using AEC as chromogne. Scale bars on (A–C) were 50µm. Scale bar on (D) was 100µm. [file Image1.tif]
